# Monitoring continuity of maternal and child health services, Indonesia

**DOI:** 10.2471/BLT.21.286636

**Published:** 2021-12-29

**Authors:** Siti Helmyati, Dhian P Dipo, Insan Rekso Adiwibowo, Maria Wigati, Erri Larene Safika, Muhammad Hafizh Hariawan, Monita Destiwi, Yoga Prajanta, Mirza HST Penggalih, Toto Sudargo, Dewi MD Herawati, Tiara Marthias, Masrul Masrul, Laksono Trisnantoro

**Affiliations:** a Department of Nutrition and Health, Universitas Gadjah Mada, Yogyakarta, 55281, Indonesia.; b Directorate of Public Health Nutrition, Ministry of Health, Jakarta, Indonesia.; c Center for Health Policy and Management, Universitas Gadjah Mada, Yogyakarta, Indonesia.; d Center for Health and Human Nutrition, Universitas Gadjah Mada, Yogyakarta, Indonesia.; e Department of Public Health, Padjadjaran University, Bandung, Indonesia.; f Department of Health Policy and Management, Universitas Gadjah Mada, Yogyakarta, Indonesia.; g Department of Nutrition, Andalas University, Padang, Indonesia.; Correspondence to Siti Helmyati (email: siti.helmyati@gmail.com).

## Abstract

**Objective:**

To implement an online system to evaluate the impact of the coronavirus disease 2019 (COVID-19) pandemic on maternal and child health and nutrition essential health services in Indonesia.

**Methods:**

We developed an electronic monitoring and evaluation system to assist district health offices in making rapid assessments of the impact of COVID-19 on maternal and child health and nutrition programmes in their area and in developing policy and programme responses. This implementation research was conducted from September to December 2020 in 304 districts. The strategies consisted of technical assistance for district offices by 21 partner universities and development of an online dashboard for rapid situation analyses and reporting. We collected qualitative data on feasibility and adherence to the intervention, as well as quantitative data from routine health databases to analyse the impact of COVID-19 on maternal and child health and nutrition indicators.

**Findings:**

In the majority of districts key maternal and child health and nutrition services were moderately or severely affected by the pandemic, particularly child growth monitoring and antenatal care services. Adherence to the protocol of the intervention varied across districts but the system is a feasible approach to be scaled up to other regions and health programmes. High uptake by the health ministry, district office and university partners provided the platform with collaborative efforts for health-systems strengthening.

**Conclusion:**

The electronic monitoring and evaluation system could be implemented and completed with several modifications to accommodate district offices and universities. There is a potential to scale up the intervention with better implementation planning and training.

## Résumé

**Objectif:**

Instaurer un système en ligne pour évaluer l'impact de la pandémie de maladie à coronavirus 2019 (COVID-19) sur les services essentiels de nutrition et de santé maternelle et infantile en Indonésie.

**Méthodes:**

Nous avons mis au point un système électronique de surveillance et d'évaluation afin d'aider les bureaux de la santé des districts à identifier rapidement les effets de la COVID-19 sur les programmes de nutrition et de santé maternelle et infantile dans leur région, et à déployer des politiques et programmes capables de les contrer. Cette étude de mise en œuvre a été menée de septembre à décembre 2020 dans 304 districts. Les stratégies prévoyaient de fournir une assistance technique aux bureaux de district avec le concours de 21 universités partenaires, et de créer un tableau de bord en ligne servant à établir rapidement des analyses et rapports de situation. Nous avons récolté des données qualitatives sur la faisabilité et l'adhésion à l'intervention, ainsi que des données quantitatives issues des bases de données sur les soins de routine, en vue d'analyser l'impact de la COVID-19 sur les indicateurs de nutrition et de santé maternelle et infantile.

**Résultats:**

Dans la majorité des districts, les principaux services de nutrition et de santé maternelle et infantile ont été modérément ou gravement touchés par la pandémie, en particulier le suivi de la croissance des enfants et les soins prénatals. Bien que le respect du protocole d'intervention ait varié d'un district à l'autre, le système constitue une approche envisageable pouvant être étendue à d'autres régions et programmes de santé. La participation active et les efforts conjoints du ministère de la Santé, des bureaux de district et des partenaires universitaires ont permis à la plateforme de renforcer les systèmes de santé.

**Conclusion:**

Le système électronique de surveillance et d'évaluation pourrait être appliqué et accompagné de plusieurs modifications pour s'adapter aux différents bureaux de district et universités. Cette intervention peut être amenée à se développer en améliorant la formation et la planification de la mise en œuvre.

## Resumen

**Objetivo:**

Aplicar un sistema en línea para evaluar los efectos de la pandemia de la coronavirosis de 2019 (COVID-19) en los servicios sanitarios esenciales de salud y nutrición maternoinfantil en Indonesia.

**Métodos:**

Se desarrolló un sistema electrónico de supervisión y de evaluación para ayudar a las oficinas distritales de salud a realizar evaluaciones rápidas sobre los efectos de la COVID-19 en los programas de salud y nutrición maternoinfantil en su zona y a elaborar respuestas normativas y programáticas. Esta investigación de aplicación se llevó a cabo de septiembre a diciembre de 2020 en 304 distritos. Las estrategias consistieron en la asistencia técnica a las oficinas de distrito a cargo de 21 universidades asociadas y el desarrollo de un tablero de mando en línea para realizar análisis rápidos de la situación y presentar informes. Se recopilaron datos cualitativos sobre la viabilidad y la adhesión a la intervención, así como datos cuantitativos de las bases de datos de salud sistemáticas para analizar los efectos de la COVID-19 en los indicadores de salud y nutrición maternoinfantil.

**Resultados:**

En la mayoría de los distritos, los servicios clave de salud y nutrición maternoinfantil se vieron moderada o gravemente afectados como consecuencia de la pandemia, en especial la supervisión del crecimiento infantil y los servicios de asistencia prenatal. La adhesión al protocolo de la intervención varió en los diferentes distritos, pero el sistema es un método viable que se puede ampliar a otras regiones y programas sanitarios. La participación activa del ministerio de sanidad, la oficina del distrito y los socios universitarios proporcionó una plataforma de colaboración para el fortalecimiento de los sistemas sanitarios.

**Conclusión:**

El sistema electrónico de supervisión y de evaluación podría aplicarse y completarse con varias modificaciones que se adapten a las oficinas de distrito y a las universidades. Existe la posibilidad de ampliar la intervención mediante la mejora de la formación y la planificación de la aplicación.

## ملخص

**الغرض:**

تنفيذ نظام عبر الإنترنت لتقييم تأثير جائحة مرض فيروس كورونا 2019 (كوفيد 19) على صحة كل من الأم والطفل، وخدمات التغذية الصحية الأساسية في إندونيسيا.

**الطريقة:**

قمنا بتطوير نظام إلكتروني للمراقبة والتقييم، لمساعدة مكاتب الصحة في المقاطعات في إجراء تقييمات سريعة لتأثير كوفيد 19 على صحة كل من الأم والطفل، وبرامج التغذية في منطقتهم، وفي تطوير الاستجابات للسياسات والبرامج. تم إجراء بحث التنفيذ هذا من سبتمبر/أيلول إلى ديسمبر/كانون أول 2020 في 304 منطقة. تكونت الاستراتيجيات من المساعدة الفنية لمكاتب المقاطعات بواسطة 21 جامعة شريكة، وتطوير لوحة معلومات عبر الإنترنت لتحليل المواقف وإعداد التقارير على وجه السرعة. قمنا بجمع بيانات نوعية حول الجدوى من التدخل والالتزام به، بالإضافة إلى بيانات كمية من قواعد البيانات الصحية الروتينية لتحليل تأثير كوفيد 19 على صحة كل من الأم والطفل، وكذلك مؤشرات التغذية.

**النتائج:**

في غالبية المناطق، تأثرت خدمات صحة الأم والطفل الرئيسية وخدمات التغذية بشكل معتدل أو شديد نتيجة الجائحة، ولا سيما متابعة نمو الطفل وخدمات الرعاية السابقة للولادة. تنوع التقيد ببروتوكول التدخل عبر المناطق، ولكن النظام يُعد أسلوبًا عمليًا لتوسيع نطاق التدخل ليشمل المناطق والبرامج الصحية الأخرى. إن الاستيعاب الكبير بواسطة وزارة الصحة، ومكتب المنطقة، والشركاء من الجامعات، أدى إلى توفير المنصة بجهود تعاونية لتعزيز النظم الصحية.

**الاستنتاج:**

يمكن تنفيذ النظام الإلكتروني للمراقبة والتقييم وإكماله مع العديد من التعديلات لاستيعاب مكاتب المقاطعات والجامعات. هناك إمكانية لتوسيع نطاق التدخل من خلال تنفيذ أفضل للتخطيط والتدريب.

## 摘要

**目的:**

印度尼西亚实施电子系统来评估 2019 年冠状病毒病 (COVID-19) 疫情对母婴健康和营养基本卫生服务的影响。

**方法:**

我们开发了一个电子监测和评估系统，以协助地区卫生办公室快速评估新冠肺炎对其所在地区母婴健康和营养计划的影响，并制定相关政策和计划响应。该项研究于 2020 年 9 月至 12 月在 304 个地区实施。这些战略包括由 21 所合作大学为地区办公室提供技术援助，和开发用于快速分析和报告情况的在线仪表板。我们收集了有关干预措施的可行性和依从性的定性数据，以及来自常规健康数据库的定量数据，来分析新冠肺炎对母婴健康和营养指标的影响。

**结果:**

在大多数地区，重要的母婴健康和营养服务，特别是儿童生长监测和产前护理服务，受到疫情的中度或严重影响。各地区对干预方案的遵守情况各不相同，但该系统是一种可行的方法，可以推广到其他地区和卫生计划。卫生部、地区办公室和大学合作伙伴的高度接受，为该平台提供了加强卫生系统的合作努力。

**结论:**

电子监测和评估系统可以在进行修改后进行实施和完成，以便适应地区办公室和大学。可以通过更好的实施规划和培训，来扩大干预范围。

## Резюме

**Цель:**

Внедрить онлайновую систему для оценки влияния пандемии коронавирусной инфекции 2019 года (COVID-19) на основные услуги в области охраны здоровья и обеспечения питания матери и ребенка в Индонезии.

**Методы:**

Авторы разработали электронную систему мониторинга и оценки, чтобы помочь районным отделениям здравоохранения быстро оценить влияние COVID-19 на программы в области охраны здоровья и обеспечения питания матери и ребенка в своем регионе, а также разработать политику и программные меры реагирования. Такое имплементационное исследование проводилось с сентября по декабрь 2020 года в 304 районах. Стратегии включали техническую помощь районным отделениям со стороны 21 университета-партнера и разработку онлайновой информационной панели для быстрого анализа ситуации и отчетности. Были собраны качественные данные о целесообразности и последовательном исполнении данного мероприятия, а также количественные данные из стандартных баз данных о здравоохранении для анализа влияния COVID-19 на показатели состояния здоровья и питания матери и ребенка.

**Результаты:**

В большинстве районов основные услуги в области охраны здоровья и обеспечения питания матери и ребенка пострадали от пандемии в умеренной или серьезной степени, особенно услуги по мониторингу развития детей и дородовой помощи. Соблюдение протокола мероприятия разнилось в зависимости от района, но эта система представляет собой вполне реализуемый подход, который можно внедрять в других регионах и программах здравоохранения. Высокая популярность платформы на уровне Министерства здравоохранения, районного отделения и университетов-партнеров послужила основой для сотрудничества по укреплению систем здравоохранения.

**Вывод:**

Электронную систему мониторинга и оценки можно внедрять, а также можно вносить в нее изменения с учетом районных отделений и университетов. Существуют возможности для расширения масштабов мероприятия за счет лучшего планирования его внедрения и подготовки кадров.

## Introduction

Indonesia, a lower-middle-income country with a population of more than 260 million people, has a high burden of infant and maternal mortality and child undernutrition. In 2018, the prevalence of stunting was 30.8% (27 023 of 87 737 children aged 0–59 months),^1^ significantly higher than in other countries with comparable economic development status.^2^^,^^3^ The prevalence also varies widely across provinces, ranging from 17.7% in the capital city Jakarta to 42.6% in the less-developed province of East Nusa Tenggara.^1^ In 2019, Indonesia’s infant mortality (20.2 per 1000 live births) and maternal mortality (177 per 100 000 live births) were among the highest in the South-East Asia Region.^4^^–^^6^ The United Nations Children’s Fund in Indonesia has reported that lack of awareness, unequal capacity and distribution of health resources, insufficient budget allocation, and lack of vertical and intersectoral coordination were the most prominent factors related to those problems.^7^

Indonesia’s national strategy for stunting reduction has been in place since 2015.^8^^–^^10^ However, the coronavirus disease 2019 (COVID-19) pandemic has halted many public health programmes, particularly those delivered by community health centres and integrated health posts.^11^^–^^13^ By 2020, the Indonesian health system was at its capacity, with the government diverting resources towards COVID-19 mitigation.^14^^–^^16^ Budget reallocation to the pandemic has jeopardized the country’s capacity to maintain essential health services, with a notable impact on pre-existing problems,^7^ including programmes focused on the health of mothers and children’s nutrition.^11^ Providing timely data on the impact of the pandemic on routine programmes is necessary to rapidly map strategies to recover from the disruption caused by the pandemic. Managerial problems that might be faced by district health offices can provide evidence of the need for capacity improvement from external resources.

We describe our experience in developing and implementing an online system to help maintain maternal and child health and nutrition programmes in Indonesia during the COVID-19 pandemic. The initiative was a collaboration between the Ministry of Health of Indonesia and Gadjah Mada University in Special Region of Yogyakarta, with 20 other partner universities across Indonesia.

## Methods

### Study setting

This implementation research was done in provinces and islands throughout Indonesia. In 2020, the national government designated 120 districts as priority areas for reducing maternal and neonatal mortality and 260 districts as priority areas for improving nutrition, specifically child stunting. These focus areas may receive greater budget allocation from the national level for programme innovations and implementations. Since 76 districts are focus areas for both maternal and neonatal mortality and for stunting, we included a total of 304 districts in the study.

### Intervention

We developed an electronic monitoring and evaluation system (called eMonev) to assist district health offices in making rapid assessments of the impact of COVID-19 on maternal and child health and nutrition indicators in their area and in developing policy and programme responses. The health ministry appointed Gadjah Mada University as the lead academic partner to supervise and implement the system. The intervention consisted of technical assistance for district offices by partner universities and development of an online dashboard for analysis and reporting. Both strategies were designed to provide remote assistance to improve the managerial capabilities of the staff of district offices, including situation and policy analysis using routine data, and to map the impact of the pandemic and possible mitigation strategies. 

Technical assistance and mentoring to district offices was delivered by the health ministry and 21 partner universities, including the lead university. The universities were selected based on previous collaboration with the health ministry in providing expert assistance in data analysis on other projects, and each university was assigned to assist staff in 14–16 district offices closest to them. The lead university recruited all 304 district health offices to the study and held several meetings with partner universities to discuss the activities. 

We designed the online dashboard to display maps and charts summarizing the data collected. We also developed a website where participants could open the training resources, access online meetings, view other materials and upload documents written by the district health offices. The development of the dashboard and website started with gathering information and inputs from the health ministry and assessing which indicators to include. The selected indicators were routine data collected by district offices and stored in the health ministry database. Information about the impact of the pandemic, and strategies and challenges to deal with it, was gathered from the district offices and quality checked by the lead university before being transferred to the online dashboard. All activities were documented in open-access websites.^17^^–^^20^

Each district office was expected to produce three documents from an analysis of the national routine data in their area: (i) a situation or impact analysis of the pandemic on the selected indicators; (ii) a policy analysis; and (iii) a policy brief with recommendations for post-pandemic recovery. Staff at the lead university collated the reports and categorized the severity of impact of the pandemic (severe, moderate, mild, cannot be determined, or no data available) according to the number of indicators adversely affected. All documents and the results of the analysis collected by district offices were then displayed online in the dashboard.

### Implementation

**Fig. 1:**
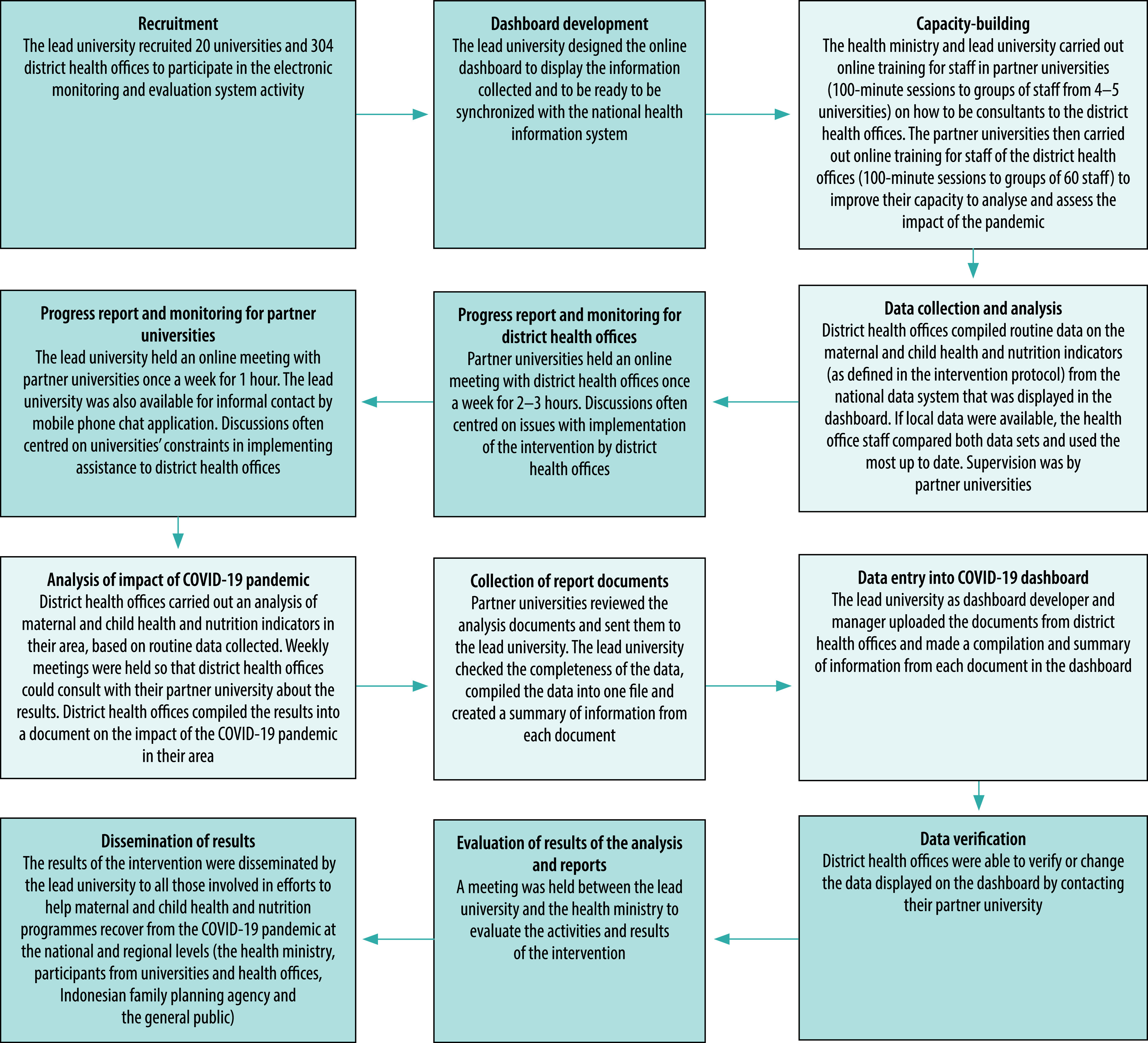
Steps in implementation of the intervention to maintain maternal and child health and nutrition programmes during the COVID-19 pandemic, Indonesia, September–December 2020

Recruitment of universities and district offices to the study began in September 2020. The intervention phase was from October to December 2020. Fig. 1 shows the steps in the implementation of the intervention from planning, recruitment and training, through data collection and analysis, to feedback to participants and dissemination to the health ministry and other stakeholders. Throughout the study we communicated with participants and collected data through online activities such as videoconferencing, email and text messages.

COVID-19: coronavirus disease 2019.

Note: Dark blue shading indicates that the step in the intervention protocol was implemented as intended; light blue shading indicates that the step was implemented with modifications.

To facilitate implementation of the intervention we designed staff development programmes for representatives of universities, to build their capacity as consultants, and for representatives of district offices, to improve their ability to analyse situations and policies. Training was delivered in online sessions by experts in the field of qualitative and quantitative data management and health management and policy. The sessions comprised an introduction to the electronic monitoring and evaluation system; training in collection and analysis of quantitative and qualitative data; and an introduction to the dashboard (more details are in Fig. 1 and the data repository).^21^ We used a blended learning approach, synchronously via videoconferencing applications and through recording material and literature on the programme website. Information on the website could be accessed by participants throughout the study. 

### Data analysis

To evaluate the implementation of the intervention we assessed the following outcomes: (i) feasibility of the intervention delivery; (ii) fidelity to the intervention protocols; (iii) availability of data for analysis; (iv) availability of documents describing the impact of the pandemic on key indicators; (v) staff development programmes received by universities and district offices; and (vi) availability of the electronic monitoring and evaluation programme’s websites (Table 1 available at: https://www.who.int/publications/journals/bulletin/). 

**Table 1:** Feasibility of the intervention to support maternal and child health and nutrition programmes during the COVID-19 pandemic, Indonesia

Implementation outcome	University supervision	Dashboard development	Data sources	Outcome
Feasibility	Feasibility of universities providing assistance to several district health offices within a certain period of time	Suitability of the online dashboard to be developed and synchronized with the national data collection system	Observations, questionnaire surveys	The programme was feasible for district health offices and universities with good internet access
Fidelity	Universities’ compliance in providing assistance to the district offices in accordance with predetermined standard operating procedures	Suitability of the online dashboard as a platform to provide data analysis and policy recommendations quickly, precisely and accurately	Observations, questionnaire surveys	Some modifications were made to accommodate the time-limitations and abilities of district health offices
Data availability	Availability of data in district offices to be analysed into information about the impact of the COVID-19 pandemic	Availability of data to display information in the online dashboard	Observations, questionnaire surveys	Not every district health office had available data in the online dashboard to be analysed
Impact of the COVID-19 pandemic on maternal and child health and nutrition services	Availability of documents prepared by the district offices, as supervised by universities, as a source of information about the impact of the pandemic on maternal and child health and nutrition services	Availability of information about the impact of the pandemic on maternal and child health and nutrition services	Observations, discussions, questionnaire surveys	The majority of district health offices were able to complete the documents for assessing the impact of the pandemic on maternal and child health and nutrition services
Staff development programmes	Type of staff development programme delivered to universities and district offices	NA	Observations, discussions	Capacity-building was provided for universities to become consultants to district health offices. Training was given to universities and district health offices on quantitative and qualitative data collection and analysis. Participants were introduced to the dashboard as the main platform of the programme
Dashboard	NA	Availability of website and online dashboard that can be used to display and find information about the impact of the pandemic on maternal and child health and nutrition services	Observations, discussions	Websites and example dashboards are available online^17^^–^^20^

COVID-19: coronavirus disease 2019; NA: not applicable.

We assessed these outcomes quantitatively by analysis of routine data on maternal and child health (based on eight indicators) and nutrition (based on six indicators) to create a descriptive summary of the extent to which each service was affected by the pandemic. We analysed the data and generated maps and charts using Excel (Microsoft Corp., Redmond, United States of America, USA), R (R Foundation for Statistical Computing, Vienna, Austria) and shinyapps (R Studio, Boston, USA) software. While data validity is a concern in the routine systems for collection of data on nutrition and maternal and child health, we aimed to use the best available data and to provide an opportunity to improve its quality via the intervention. We complemented these data with qualitative data obtained from our observations throughout the study, from discussions between universities and district offices and from questionnaires to all participants in universities and district offices. Questionnaires covered the participants’ views about implementation of the intervention, government policies related to COVID-19 and budget allocations for maternal and child health and nutrition programmes. 

## Results

### Programme analysis

More details of the participating universities and district offices are shown in the data repository.^21^ Three of the 21 universities (14%) were absent from several development meetings and did not reach all their assigned districts to assist them in completing the required documents. Of the 304 district health offices, 80 offices (26%) did not attend all the trainings and were unable to complete all the required documents. Only 158 offices (52%) were able to validate the quantitative data and provide all the necessary qualitative information. 

Adherence to standard operating procedures by participants, based on our observations, is shown by the shadings in Fig. 1. We needed to modify most of the activities in the intervention to meet the short timeline of the study, and to meet the challenge of bringing the electronic monitoring to scale in 304 districts nationwide. Our use of a blended learning system was efficient for the large number of participants and enabled participants to learn and open the materials even after the training. 

The electronic monitoring and evaluation system was effective in assisting district offices to use routine data for situation analysis and decision-making in their area (Table 1). Technical assistance by universities was an important component in helping district offices conduct these activities. Now that the national routine data are digital-based and relatively easy to use, the system has sustainability. However, challenges such as poor internet access and the small number of staff focused on maternal and child health and nutrition in the district offices and universities need to be considered (Box 1). The lead university has filed a report about these challenges to the health ministry for further action.

Box 1 **Challenges of implementing the intervention to maintain maternal and child health and nutrition programmes during the COVID-19 pandemic, Indonesia**

**Challenges reported by staff of district health offices:**

- Backlog of work at the end of the year (44 respondents);
- Small number of district office staff (27 respondents);
- Poor internet access (19 respondents);
- Rapid changes of staff of district office or community health centre (1 respondent);
- Became ill with COVID-19 (1 respondent).

**Challenges reported by staff of partner universities:**

- Negative responses from the district office staff: slow or often late in giving response, did not look interested or did not understand the aim of this study (31 respondents);
- Difficulty in coordination between university and district office or among programmes in district offices (1 respondent);
- Poor communication between head of district office and maternal and child health and nutrition implementer (1 respondent);
- Health office staff did not understand the type of data needed or how to analyse data (1 respondent);
- Prefer face-to-face monitoring and evaluation (1 respondent).

COVID-19: coronavirus disease 2019. Note: The data are only from participants who offered their comments and therefore do not represent the views of all participants.

Table 2 presents the follow-up analyses of the challenges of the pandemic reported by participants and the innovations that took place to mitigate the impact of the pandemic. We note with interest how community health workers (CHWs) coped well with the restrictions imposed by the pandemic. However, the situation varied across provinces. Several provinces would have benefited from collaboration with other ministries or agencies involved in development planning, particularly in addressing pre-existing health-system challenges. These problems include security problems (in Papua Province), lack of appropriate anthropometric measuring tools and lack of trained staff. Furthermore, many district offices reported poor internet access and infrastructure.

**Table 2:** Challenges and innovations in the implementation of the intervention to maintain maternal and child health and nutrition programmes during the COVID-19 pandemic, Indonesia, October–November 2020

Programme component	Challenges	Innovations
Growth monitoring	- Health ministry mandate to postpone services at integrated health posts from March to August 2020 (but still continue for areas with a high risk of COVID-19) led to delays in growth monitoring - Parents’ fears about COVID-19 meant that they did not want to bring their child to integrated health posts for anthropometric measurements - Civil unrest in a district in Papua Province halted activities at integrated health posts even before the COVID-19 pandemic started - Some district offices reported that integrated health posts did not have proper anthropometry gauges due to shortages of human and financial resources	- Health workers or CHWs carried out home visits to monitor children aged 0–59 months old at risk of undernutrition - Health workers created a group mobile phone chat with parents to monitor the health condition of children aged 0–59 months and share health information - Parents of children aged 0–59 months made an appointment with health workers in community health centres for anthropometry measurement - Parents who had a bodyweight scale and were able to measure body height or length at home would examine their children then report the results to the health workers by telephone
Supplementary feeding	- Some district offices reported that they had not received supplementary foods from the health ministry up to September 2020 - District offices could not provide local supplementary foods to mothers because of budget reallocation for COVID-19 mitigation and delayed disbursement of foods from the health ministry - Civil unrest in a district in Papua Province halted activities at integrated health posts even before the COVID-19 pandemic started	- CHWs made door-to-door distributions of supplementary foods for children aged 0–59 months - Health workers asked parents and their children to come to the community health centre for anthropometry measurements and receipt of supplementary foods (mostly for children aged 0–59 months with malnutrition)
Exclusive breastfeeding	- Health workers had difficulty monitoring exclusive breastfeeding since services at integrated health posts were halted and parents with newborns could not go to community health centres. Health workers had to rely on subjective answers from mothers in phone conversations about exclusive breastfeeding - Civil unrest in a district in Papua Province halted activities at integrated health posts before the COVID-19 pandemic started	- Health workers monitored the programme’s coverage using a mobile phone chat application. However, the methods could not ensure whether mothers had exclusively breastfed or not - Health workers asked mothers to come to the community health centre or vice versa when there was a nutrition counselling session. The counselling would be face-to-face, private and limited to 30 minutes
Vitamin A supplementation	- A district office in Bali Province reported that their CHWs refused to distribute vitamin A supplements door-to-door because of the risk of exposure to COVID-19 - Civil unrest in a district in Papua Province halted activities at integrated health posts even before the COVID-19 pandemic started	- Since in February 2020 no COVID-19 case was found in Indonesia and in August 2020 the cases number had lowered, the distribution of vitamin A supplements in most districts was relatively stable - Most district offices reported that health workers and CHWs were willing to distribute supplements for children aged 0–59 months door-to-door
Infant and young child feeding	- District offices reported that budget reallocation for COVID-19 mitigation had led to postponement of the infant and young child feeding programme - Several district offices from remote areas reported that they did not have trained staff in community health centres to carry out infant and young child feeding education for CHWs or mothers - Civil unrest in a district in Papua Province halted activities at integrated health posts even before the COVID-19 pandemic started	- For districts that had carried out the infant and young child feeding programme, health workers used a mobile phone chat application to communicate with CHWs and mothers and carried out online nutrition counselling sessions in groups - Community health centres that had conducted the infant and young child feeding programme could invite mothers or CHWs (in small groups or privately) to attend for a counselling session. This activity depended on the severity of COVID-19 in the areas - Health workers could give counselling on infant and young child feeding, especially for mothers of malnourished children aged 0–59 months, when they came to the community health centre for growth monitoring
Integrated management of childhood illnesses	- District offices who planned to start integrated management of childhood illness in 2020 reported that budget reallocation for COVID-19 mitigation had led to postponement of the programme - Several district offices reported they did not have trained staff in community health centres - Civil unrest in a district in Papua Province halted activities at integrated health posts even before the COVID-19 pandemic started	- Several district offices reported that, whenever possible, health workers would go to the homes of children aged 0–59 months and check their health condition - Community health centres which already had an integrated management of childhood illness service modified examination rooms to separate patients with and without signs and symptoms of respiratory illness
First antenatal care	- Some health facilities were temporarily closed due to staff shortages when staff became infected with COVID-19 - Some parents were afraid of being exposed to COVID-19 when visiting health facilities	- Telemedicine was used so that pregnant women could seek information about their pregnancy via social media managed by community health centres, mother and child clinics or health workers. Consultation with a midwife or other health worker could also be done online - Pregnant women could make an appointment in advance or register online with the health facility to avoid crowding at facilities
Maternal services coverage	- Many health staff at community health centres and hospitals were diverted to COVID-19 activities (e.g. testing, tracking and treatment), while other areas lacked staff even before the pandemic	- Pregnant women could make an appointment with the health facility for an examination to avoid crowding at facilities
Births at health care facilities	- Health ministry mandate required all mothers to have a swab PCR for COVID-19 before giving birth - Health facilities had staff shortages due to existing health workers being diverted to deal with the COVID-19 pandemic	- Health facilities could modify special delivery rooms to handle delivering mothers with COVID-19 (e.g. providing negative pressure delivery rooms to reduce the possibility of transmitting the virus and to get immediate help without having to be referred to a hospital specifically for COVID-19) - COVID-19 screening was provided for pregnant women in the third trimester
Complete neonatal visit	- Parents were afraid of exposing their children to COVID-19 when visiting health facilities	- Parents could make an appointment with the health-care facility or register their child online to get immunizations according to the schedule of the midwife or facility - Door-to-door health-care services were provided, so that health workers such as midwives could make home visits to check the health of newborns while minimizing the transmission of COVID-19
Complete basic immunization	- Parents were afraid of exposing their children to COVID-19 when visiting health facilities	- Parents could make an appointment with the health-care facility and schedule the time and duration of examination to avoid crowding at facilities
Maternal mortality number	- Some health facilities were temporarily closed. At the beginning of the pandemic, many health workers were infected with COVID-19, so health facilities that experienced a shortage of health workers chose to temporarily close until the infected health workers recovered - Some pregnant mothers were infected with COVID-19 - Some pregnant mothers were late for antenatal care appointments because health facilities were limiting the numbers of patients seen to reduce the spread of COVID-19	- Health facilities could modify special delivery rooms to handle delivering mothers with COVID-19 (e.g. providing negative pressure delivery rooms to reduce the possibility of transmitting the virus and to get immediate help without having to be referred to a hospital specifically for COVID-19) - Pregnant women could have PCR swab examination at 37 weeks of gestation at the community health centre or nearest hospital
Family planning	- Restrictions on community activities and regional movement restrictions imposed by local governments hampered the supply chain of contraceptive devices in the regions	- Women who use contraceptives such as intrauterine devices, implants or injections could contact the nearest midwife or health facility to enquire about the availability of the desired contraceptive

CHWs: community health workers; COVID-19: coronavirus disease 2019; PCR: polymerase chain reaction test.

Note: We analysed qualitative data extracted from the impact analysis documents written by district health office staff. Integrated health posts (*posyandu*) provide community-based services focusing on maternal and child nutrition such as growth and development monitoring, nutrition counselling, immunization and supplementary foods for malnourished children.

### Impact analysis

**Fig. 2:**
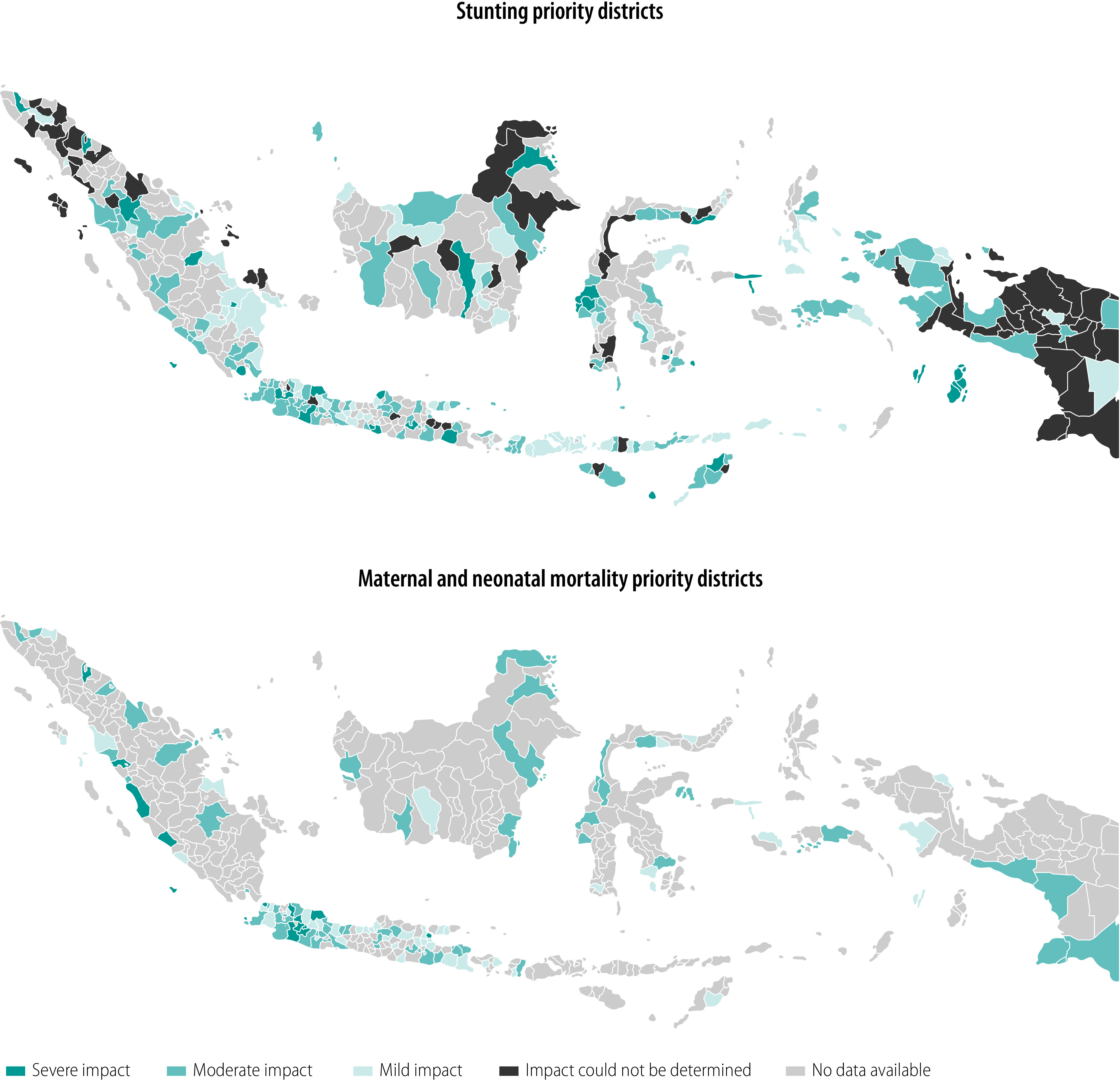
Situation map of the severity of impact of the COVID-19 pandemic on maternal and child health and nutrition programmes across Indonesian districts, 22 January 2021

Fig. 2 maps the impact of the COVID-19 pandemic on key maternal and child health and nutrition indicators across districts of Indonesia as of 22 January 2021. Based on the analyses of nutrition-related programmes in 260 priority districts, 23 districts (9%) were severely affected by the pandemic, 101 districts (39%) moderately affected, 61 districts (23%) mildly affected and 75 districts (29%) could not be assessed due to lack of data. Fig. 2 shows the impact of the pandemic on maternal and neonatal mortality indicators in 120 priority districts:15 districts (13%) were severely affected, 59 districts (49%) were moderately affected, and 46 districts (38%) were mildly affected.

COVID-19: coronavirus disease 2019.

Notes: Stunting priority districts (*n* = 260) and maternal and neonatal mortality priority districts (*n* = 120). Nutrition programmes were assessed based on six indicators: Severe impact was defined as five to six out of six indicators being negatively affected by the pandemic, Moderate Impact was three to four indicators affected, and Mild impact was one to two indicators affected. Cannot be determined was when there were insufficient reports to generate the composite index. Maternal and child health programmes were assessed based on eight indicators: Severe impact was seven to eight indicators negatively affected by the pandemic, Moderate impact was four to six indicators affected, and Mild impact was one to three indicators affected.

**Fig. 3:**
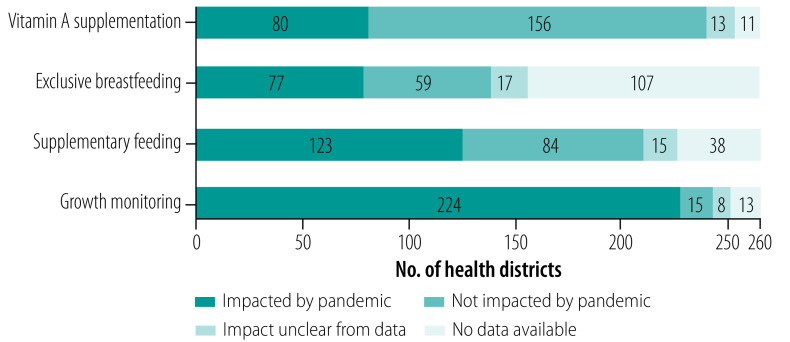
Impact analysis of the overall severity of impact of the COVID-19 pandemic on key nutrition indicators across 260 priority districts, Indonesia, 22 January 2021

**Fig. 4:**
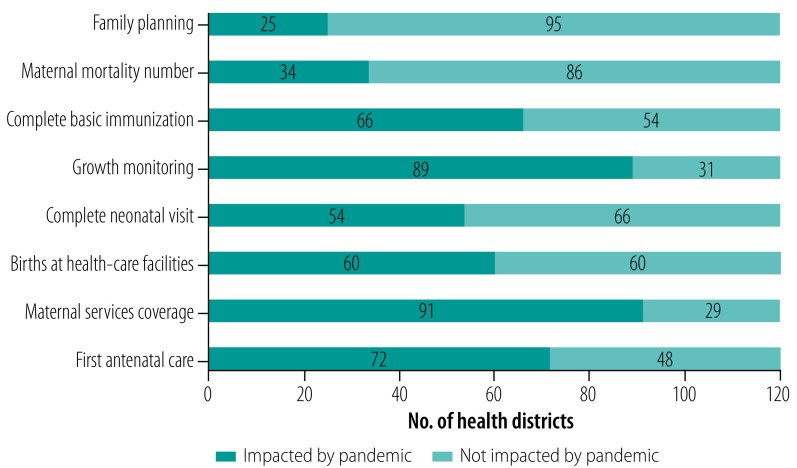
Impact analysis of the overall severity of impact of the COVID-19 pandemic on key maternal and child health indicators across 120 priority districts, Indonesia, 22 January 2021

We also assessed the impact of the COVID-19 pandemic on four of the six nutrition indicators (Fig. 3) and eight maternal and child health indicators (Fig. 4) as of 22 January 2021. Growth monitoring (nutrition indicators) and maternal services coverage (at least four visits; maternal and child health indicators) were the most affected (89 and 91 districts affected, respectively), while vitamin A supplementation (nutrition indicators) and family planning services (maternal and child health indicators) were the least affected (80 and 25 districts, respectively).

COVID-19: coronavirus disease-2019.

Notes: Nutrition services were assessed based on six indicators but the chart shows only the four indicators measured by quantitative methods; the other two indicators were based on subjective opinions from the district health offices. Severe impact was defined as five to six out of six indicators being negatively affected by the pandemic, Moderate impact was three to four indicators affected, and Mild impact was one to two indicators affected. We also made a note of districts where there were insufficient reports to generate the composite index. The numbers shown are the number of districts. For nutrition programmes, Impacted means that the programme’s coverage in the district was negatively impacted by the COVID-19 pandemic marked by a decrease of the coverage during the pandemic. Not impacted means that the programme’s coverage was not negatively impacted marked by the stable coverage during the pandemic. Impact unclear from data means the data and information provided was ambiguous so no conclusions could be drawn.

COVID-19: coronavirus disease 2019.

Notes: Maternal and child services were assessed based on eight indicators: Severely affected was seven to eight indicators negatively affected by the pandemic, Moderate impact was four to six indicators affected, and Mild impact was if one to three indicators were affected. The numbers shown are the number of districts. For maternal and child health programmes, Impacted means that the programme’s coverage was negatively impacted by the COVID-19 pandemic marked by a decrease of the coverage during the pandemic. Not Impacted means that the programme’s coverage was not negatively impacted marked by stable coverage of the programme during the pandemic.

### Policy analysis

The impact analysis was followed by policy analysis and recommendations for strategies to support maternal and child health and nutrition programmes during the pandemic (Box 2). The most prominent follow-up actions suggested by district offices and their partner university were improving the health information system; engaging the community for action on maternal and child health and nutrition programmes; and solving underlying health-system problems such as lack of staff and insufficient anthropometric measuring tools in field offices.

Box 2 **Strategies to support maternal and child health and nutrition programmes during the COVID-19 pandemic, Indonesia**

**Adjusting to COVID-19 situation**

- Using information technology
- Adding appropriate anthropometric measuring tools
- Adding system for patients to book health centre appointments or home visits
- Adding more protective equipment against COVID-19

**Capacity-building**

- Adding staff if needed
- Providing assistance for field officers
- Providing continued assistance for the district or provincial health offices

**Strengthening intersectoral coordination**

- Increasing interagency commitment to reducing the prevalence of maternal and child health and nutrition problems (e.g. stunting and maternal and neonatal deaths)
- Ensuring that nutrition and health programmes can be carried out safely in conflict-affected areas

**Strengthening management system**

- Increasing the role of the national routine databases as the basis for decision-making
- Adding information technology staff if necessary

**Potential partners**

- Health ministry
- Provincial health offices
- District health offices
- Provincial or district informatics and technology offices
- Leaders of districts or provinces
- Department of public works and public housing
- District or city food security service
- Community leaders, traditional leaders, religious leaders

COVID-19: coronavirus disease 2019.

## Discussion

We present our experience in developing an approach to monitoring the impact of the COVID-19 pandemic on key components of the routine health-care system in Indonesia.^22^ The intervention allowed for rapid situation assessments of the impact of the pandemic on mother and child and nutrition programmes in individual districts and nationwide. The use of digital technology was effective and time-efficient – factors which are important in settings such as Indonesia with a large population and diverse geographical conditions, and during external shocks such as a pandemic.

We found evidence of good feasibility of the online system. The health ministry fully supported using digital-based routine data for monitoring essential services, and strengthened the platforms by further online training for district offices after the required activities had been completed. The intervention is in line with World Health Organization’s recommendation to use routine data to maintain essential health services.^22^ The routine collection of nutrition and maternal and child health indicators are now digital-based and easier to use and access. University staff found the online system easier to operate and more efficient than paper-based and face-to-face methods, especially after much of the resources of district offices were diverted to managing the COVID-19 pandemic. Furthermore, the system was considered more convenient for universities, particularly during travel restrictions. University partners were enthusiastic in assisting the district offices, in line with the culture of research and community service in higher education in Indonesia.^23^ District offices recognized the important role of universities in improving their ability to analyse data and make policy recommendations, and the majority of them still communicated with universities after the study ended. 

There were challenges, however. Fidelity to the intervention in terms of adherence to standard protocols by district office and university staff showed some weaknesses. A major component of the intervention was the analysis of national routine data that were available on the dashboard and were easy to access online for district offices. However, several district officers preferred to use their own data for analysis since they believed that these were more complete, up to date and reliable than the data in the national systems which were collected by CHWs at integrated health posts who were not properly trained. Furthermore, data were not regularly updated by the community health centre or district offices due to limitations in human resources, infrastructure and internet access. These factors adversely affected the completion of the intervention activities by district offices, even with supervision and encouragement from their partner university. District offices around the more remote eastern regions and small islands of Indonesia were especially affected. Poor adherence to the intervention protocols highlighted gaps in resources across Indonesia. Furthermore, several district offices reported that staff were reassigned to handling the COVID-19 pandemic, and therefore fewer staff were available to manage maternal and child health and nutrition programmes. High workloads and administrative duties also hindered the full participation of district offices in the intervention activities. 

We made several modifications to accommodate the time-limitations and the abilities of district offices since this was their first experience with electronic monitoring and evaluation. Despite the circumstances, partner universities continued to assist and supervise district offices via online meetings, messages and phone calls and to use available routine health data for analysis. Universities emphasized the importance of completing the activities in the protocol and the benefits to district offices if they were able to complete all the necessary documents for display on the dashboard, including gaining support from the health ministry and stakeholders. Nevertheless, several district offices could not complete all the activities in the given time. 

The biggest challenge to effective implementation of the intervention was the inequalities in resources across district offices and universities. Several district offices have few human resources focusing on maternal and child health and nutrition programmes, and some offices struggled to adjust to an online-based work system. Furthermore, mobile phone and internet networks are not equally available across Indonesia, causing difficulties for several participants. University staff too were often unfamiliar with the data and how the platforms worked, so additional training was needed. Some university staff were newly assigned to the district offices, hence training for a consultant role is continuously needed. Better planning and leadership from the health ministry is needed for the intervention to be more effective.

Another weakness of the intervention was the uneven geographical distribution of partner universities, which were mainly from highly populated Java Island. To better assist the district offices, a more diverse selection of universities need to be engaged in the programme. We also noted that district offices in geographical proximity to universities were more active and had better understanding about the COVID-19 impact in their area.

The dashboard can rapidly display the status of essential services across regions or over time, which helped in analysing the situation for further recommendations to relevant stakeholders. After the dissemination of the results of the monitoring and evaluation at the end of 2020, there was a discussion among the health ministry, universities and family planning agency about synchronizing the dashboards to the routine national data collection platforms for nutrition and maternal and child health indicators. Unfortunately, the plan was postponed due to limited funds from budget reallocation for handling COVID-19. The health ministry is now focusing on strengthening routine data use in community health centres and district or provincial health offices. 

The implementation was funded by the health ministry at a total cost of 703.6 million Indonesian rupiahs, or 49 645 United States dollars (US$). The funding was split into six categories: implementation design, development of programmes, training, analysis and reporting, supervision of universities, and universities’ fees, with the last category receiving the largest share of US$ 42 333. Given the large number of district offices and universities involved and the positive outcomes of the system, we believe that the cost of the intervention was reasonable.

This study has some limitations. First, the evaluations of fidelity and feasibility were limited. We could only identify participants’ adherence to standard protocols. Moreover, our primary measure of adherence and exposure were observations and questionnaire surveys, although the latter could not reach all participants. We did not evaluate district offices on the quality of training and development activities they received, the professional supervision by universities or the convenience of the dashboard. The information we gathered was not measured through direct surveys or evaluations. Second, there was no comparison group to compare the quality and delivery of training and supervision. Third, the duration of the monitoring and evaluation from planning to implementation was short, only 3 months, and operated at the end of the year when district office staff reported a backlog of work. The short timescale of the intervention likely influenced the willingness of district offices and universities to complete the programme.

The implementation of the intervention has had some positive outcomes. First, the health ministry, universities, district health offices and other stakeholders have begun to appreciate the benefit of routine health data and use of information technology systems for rapid health system assessment. Routine health data also avoid the need for and reliance on costly health surveys. Participants understood the importance of data quality and validity and the efforts needed to improve the health information system. There is now an established network between universities and district offices managing maternal and child health and nutrition programmes, including data validation, which could lead to better collaboration to improve the quality of services delivery in the future.

We also observed a positive longer-term impact following the intervention. In 2021, the health ministry began using training activities to further scale up the use of national health routine data for decision-making by district offices and started collaborating with various universities to strengthen the routine data collection systems for maternal and child health and nutrition indicators. The national population and family planning agency, an agency mandated to reduce stunting prevalence in Indonesia, is now planning to cooperate with universities to assist districts in implementing, monitoring and evaluating stunting reduction programmes.

Data integration would be important to improve maternal and child health and nutrition programmes as many of the variables are collected by different units within the district offices or by external agencies. This would allow for more rapid analysis and programme planning or recommendations.

The implementation of the electronic monitoring and evaluation has offered the potential for better use of routine health data in programme monitoring and evaluation. The online platform allows for better coverage across countries with vast geographical areas and large populations. While several modifications and continuous improvement are pertinent, we propose that a similar approach could be scaled up to other programmes and beyond the pandemic period.
